# Juglone induces ferroptosis in glioblastoma cells by inhibiting the Nrf2-GPX4 axis through the phosphorylation of p38MAPK

**DOI:** 10.1186/s13020-024-00920-2

**Published:** 2024-03-22

**Authors:** Fangzhou Guo, Guoyuan Ling, Jianting Qiu, Jicheng Li, Yu Gan, YingYing Yu, Jiamei Tang, Ligen Mo, Haozhe Piao

**Affiliations:** 1 Department of Neurosurgery, Guangxi Medical University Cancer Hospital, No. 71 Hedi Road, Nanning, 530021 Guangxi China; 2 Graduate School, Dalian Medical University, Dalian, 116044 Liaoning China; 3 Department of Neurosurgery, Cancer Hospital of China Medical University, Cancer Hospital of Dalian University of Technology, No.44 Xiaoheyan Road, Dadong District, Shenyang, 110801 Liaoning China; 4 Graduate School, China Medical University, Shenyang, 110042 Liaoning China; 5 Graduate School, Liaoning University of Traditional Chinese Medicine, Shenyang, 110042 Liaoning China

**Keywords:** Juglone, Glioblastoma, Ferroptosis, p38MAPK, Nrf2

## Abstract

**Background:**

Ferroptosis, a non-apoptotic form of cell death induced by accumulation of free iron ions and lipid peroxidation, its importance for cancer treatment is gradually being recognized. Research on the anti-cancer mechanism of juglone is accumulating. However, the specific mechanism by which it directs glioblastoma (GBM) to death is unknown.

**Methods:**

We used in vitro and in vivo experiments to explore the anti-GBM effect generated by juglone through the ferroptosis pathway.

**Results:**

Juglone mainly causes cell death by inducing ferroptosis. Mechanistically, juglone can significantly activate the phosphorylation of p38MAPK. According to transcriptome sequencing and protein interaction analysis, the Nrf2-GPX4 signaling pathway is identified as the primary pathway through which juglone mediates ferroptosis. In vitro and in vivo experiments further verified that juglone induces the ferroptosis of GBM by activating the phosphorylation of p38MAPK and negatively regulating the Nrf2-GPX4 signaling pathway.

**Conclusion:**

Juglone induces ferroptosis and inhibits the growth of GBM by targeting the Nrf2/Gpx4 signaling pathway and thus holds promise as a novel ferroptosis inducer or anti-GBM drug.

**Supplementary Information:**

The online version contains supplementary material available at 10.1186/s13020-024-00920-2.

## Introduction

Glioblastoma (GBM) is the most common primary malignant intracranial tumor with a very low 5-year survival rate, less than 3% for primary GBM and less than 10% for secondary GBM [1, 2]. GBM are highly malignant and invasive, and cannot be completely removed by surgery. The existing treatments mainly consist of maximized surgical resection followed by radiotherapy and chemotherapy to extend survival [3, 4]. Temozolomide is a DNA alkylating agent that has been used as a first-line chemotherapy drug for GBM since 2005, but resistance to temozolomide is common in patients with tumor recurrence or those without MGMT promoter methylation, leading to treatment failure [5, 6]. In recent years, electrical field treatment may benefit some postoperative patients [7]. Another FDA-approved clinical treatment program includes the anti-angiogenesis targeted drug—Bevacizumab, which can improve the quality of life for some patients, however, the overall survival has not benefited [7]. Other treatments include BRAF p.V600E mutation inhibitors dabrafinib/trametinib, ALK gene fusion targeting drug Alectinib, CDK4/6 inhibitors, mTOR pathway inhibitors everolimus, and anti-PD-1 treatment have all been actively tried, however, the therapeutic effects are not satisfactory [8–11]. In order to overcome such limitations of treatments, research focus has shifted towards finding new death pathways to control tumor growth [12, 13].

Ferroptosis represents a unique form of cell death, distinct from necrosis and apoptosis, that relies on the presence of intracellular iron ions. It is characterized by the accumulation of lipid peroxides that involve iron ions [14, 15]. Iron-induced cell death has been observed for several decades; however, it was not formally identified as ‘ferroptosis’ until 2012 when Dixon et al. discovered that the small- molecule erastin activated an unconventional death pathway in RAS mutant carcinoma cells [16]. Rapidly dividing cancer cells require large quantities of iron ions, making them particularly susceptible to ferroptosis due to their iron dependence [17]. As a result, the induction of ferroptosis has emerged as an innovative therapeutic strategy [18, 19]. Increasing research provides evidence that evading ferroptosis can enhance the invasiveness and resistance of GBM, whereas inhibiting the ferroptosis pathway can decrease its immune evasion [20, 21]. Therefore, clinical research focused on ferroptosis could potentially improve the treatment outcomes of GBM.

Juglone, scientifically known as 5-hydroxy-1,4-naphthoquinone, is a natural naphthoquinone compound extracted from the immature outer fruit skin (green skin) of plants from the Juglandaceae family, namely walnut and its related species black walnut [22]. Early research revealed its inhibitory effect on Pin1 activity and the potential to induce tumor apoptosis [23], and it also has a positive effect on traditional medicinal effects such as promoting blood circulation, removing wind and treating ringworm, and clearing heat and detoxification [24]. Recent research has further discovered that juglone has a wide range of anti-cancer effects. For example, juglone can delay DNA repair, inhibit the proliferation of melanoma cells, and also stop the expression of ACC1 protein to inhibit the proliferation of cancer cells [25]. Recent studies have revealed that juglone may inhibit the growth of endometrial cancer and pancreatic cancer through the ferroptosis pathway [26, 27], and research has found that juglone has a broad spectrum of anti-tumor activity, and due to its small molecular weight and lipid solubility, it can easily penetrate the blood–brain barrier, which may have unique advantages in the treatment of gliomas. Studies have confirmed that it can exert its anti-glioma effect in both in vitro and in vivo in human GBM cells and rat C6 glioma cells. Potential mechanisms include promoting the activity of the MAPK family, inducing apoptosis by activating the caspase cascade reaction, and affecting the intracellular oxidative stress system [28, 29]. However, the specific mechanism of juglone inducing GBM cell death has not been fully studied, especially whether it inhibits tumor cell proliferation through the ferroptosis pathway. Therefore, we will explore whether the ferroptosis pathway is involved in the anti-GBM characteristics of juglone.

Nrf2 is a key transcription factor within cells, capable of regulating a multitude of antioxidant enzymes, thereby playing a core role in the antioxidant defense system. It is involved in diverse pathways, rendering its functionality fairly complex. Recent research indicates that Nrf2 exhibits high expression in both GBM cell lines and GBM tumor tissues [30–32]. Furthermore, the expression of Nrf2 in the cell plasma is associated with poor prognosis in GBM patients [31]. One study found that downregulating Nrf2 resulted in an increase in apoptotic factors (such as caspase, Bcl-2, HO-1, etc.), making GBM cells more susceptible to apoptosis [33]. Another study found that there is a synergistic regulation feedback between autophagy regulatory protein complex Sequestosome1 (SQSTM1/p62) and Nrf2, which aids in driving the stromal phenotype of GBM cells [34]. Additionally, research shows that inhibiting the expression of Nrf2 and its targeted proteins in GBM cell lines can enhance the sensitivity of GBM cells to Temozolomide [35]. Therefore, the importance of Nrf2 in the GBM pathway is unquestionable, and it can be viewed as a potential therapeutic target. A thorough investigation into the interactions between Nrf2 and the key molecular signaling mechanisms in GBM cells may provide novel insights for GBM treatment.

This study for the first time demonstrated that juglone inhibits the growth of GBM cells in vivo and in vitro by promoting ferroptosis through the negative regulation of the Nrf2/GPX4 axis by elevating the phosphorylation level of p38MAPK. This provides a new anti-GBM mechanism, suggesting that juglone may potentially be an anti-GBM therapeutic strategy.

## Materials and methods

### Cell culture

LN229 and T98G cell lines were obtained from American Type Culture Collection (ATCC) and maintained in DMEM culture medium (BasalMedia, China) containing 5% and 10% fetal bovine serum and 1% penicillin/streptomycin, respectively. The culture temperature was 37 °C, and the CO2 concentration was 5%. The cells came from our laboratory stocks and, after resuscitation, these cells were used for no more than 10 generations.

### Antibodies and reagents

Juglone, Chloroquine (CQ-1), Ferrostatain-1 (Fer-1), Liproxstatin-1 (Lip-1), Mdivi-1 (Mdivi), Necrostatin-1 (Nec-1) and Z-VAD-FMK (Z-VAD) were purchased from Selleck. 2ʹ,7ʹ-Dichlorodihydrofluorescein diacetate (DCFDA) were purchased from MCE. Antibodies against ACSL4, PTGS2, GPX4, Ki67, 4HNE were purchased from Abcam. Antibodies against XCT, FTH1, p38, pp38, JNK, p-JNK, GSK3α/β, p-GSK3α/β, MMp2 and MMp9 were purchased from Cell Signal Technology. Antibodies against TFRC were purchased from Invitrogen. Antibodies against α-Tubulin and GAPDH, as well as secondary antibodies, were purchased from Hua-an Biotechnology. Details and information of antibodies are provided in Additional file 1: Table S1.

### Cell viability assay

Approximately 3000–5000 cells were inoculated into each well of a 96-well plate. After 24 h of treatment with different concentrations of juglone with or without inhibitor, the supernatant was discarded, 100 μL of DMEM medium containing 10% CCK-8 (APE × BIO, K1018) was added to each well, incubated for 1 h at 37 °C, then the absorbance was measured at a wavelength of 450 nm.

### Colony formation assessment

Approximately 1500–2000 cells per well were inoculated in a 6-well plate. Until the cells grew into visible colonies. Then LN229 or T98G cell lines were maintained in juglone medium for 24 h (cells pre-treated with inhibitor for 1 h), then the medium was replaced with normal growth medium. The same operation needed to be repeated approximately 2 times, with the cells maintained for about 15 days. Fixed with 4% polyformaldehyde for 30 min, stained with crystal violet solution (1% crystal violet in 95% ethanol) for 2 h.

### Western blotting analysis

RIPA buffer supplemented with protease inhibitors was used to extract total protein; protein quantitation was conducted using the BCA protein assay kit (Biosharp, China). Samples were electrophoresed on a 10% SDS polyacrylamide gel (SDS-PAGE), then were transferred onto an NC membrane. The membrane was then blocked with 5% non-fat milk for 1 h, followed by washing, incubation with primary and secondary antibodies, and finally detected with enhanced chemiluminescence. The Nuclear and Cytoplasmic Protein Extraction Kit (78833, Thermo Scientific) was used for nuclear Nrf2 and cytosol Nrf2 extraction according to the manufacturer’s instructions, GAPDH and histone H3 (1:3000; Abcam) were served as internal reference proteins.

### Immunofluorescence assay

LN229 and T98G cells treated with different concentrations of Juglone for 24 h were fixed with 4% polyformaldehyde for 15 min. After fixation, the cells were washed twice with PBS and treated with PBS solution of 0.2% Triton-X 100 for 15 min. Then, the cells were incubated with 5% skim milk powder at room temperature for 1 h. Primary antibody was incubated at 4 °C for 12 h, and the secondary antibody was incubated at room temperature for 1 h, DAPI staining was done for 10 min, followed by the addition of anti-fluorescence quenching agents and observed with a fluorescence detection microscope. Images were captured and analyzed subsequently with the MetaView software.

### Immunoprecipitation assay

After the cells were treated with different concentrations of juglone for 24 h, the cells were collected, lysed with a lysis buffer to obtain the supernatant, and centrifuged to obtain the supernatant, followed by overnight incubation at 4 °C with 2 μL of the antibody and 20 μL of the Protein A/G beads (Thermo Scientific). The sample was then centrifuged, washed three times with IP buffer, then the resulting protein sample was resuspended in 1 × loading buffer and subjected to a metal bath at 100 °C for 10 min, and the immunoprecipitated protein was analyzed by immunoblotting with anti-ubiquitin antibody.

### Intracellular reactive oxygen species (ROS) determination

Approximately 2 × 10^5^ cells were seeded onto 6-well plates. After 24 h of incubation, LN229 and T98 cells were treated with different methods for another 24 h, then replaced with HBSS containing 10 μmol/L DCFDA for incubation for 30 min. The cells were washed twice with PBS to remove DCFDA. Then the cells were resuspended in 500 μL HBSS and detected by flow cytometry, and the results are analyzed using FlowJo V10 software. Meanwhile, DCFDA was also used for immunofluorescence detection. LN229 and T98 cells were seeded in 6-well plate and subjected to different treatments for 24 h. Then they were stained with 10 μmol/L DCFDA for 1 h and then stained with Hoechst for 10 min. The fluorescence microscope was used to analyse the results.

### Glutathione (GSH) and malonaldehyde (MDA) levels were measured

The measurements of intracellular GSH and MDA levels were determined using GSH test kit (G263, Tong Ren, Japan) and MDA test kit (Sangon, China) respectively. All experimental operations were completed according to the kit operating instructions.

### Potential targets prediction and screening of juglone

The ferroptosis-related genes were sourced from the FerrDb database (http://www.zhounan.org/ferrdb) [36]. The potential targets of juglone were predicted using Swiss Target Prediction (http://www.swisstargetprediction.ch/) by applying the downloaded Juglone’s SDF and mol2 format [37]. The protein–protein interaction (PPI) network was obtained from the STRING database 11.0 (http://string-db.org/) and was analyzed and visualized through Cytoscape 2.8.2 [38]. Further, the R language software was used to analyze GO and KEGG data and draw volcano plot and heatmaps.

### Molecular docking was performed to predict the interaction pattern

The crystal structure corresponding to the Keap1 and p38 proteins was obtained from the RCSB PDB database (Structural Bioinformatics Protein Data Bank) [39]. The protein crystals obtained were processed separately using the Protein Preparation Wizard module in Schrödinger software. The 2D structure data file of the compound juglone was processed using the LigPrep module in Schrödinger to generate all its 3D chiral conformations. The prepared ligand compound Juglone was docked with the active sites of the p38 and Keap1 protein structures, respectively, and the scores were calculated.

### Construction of overexpression plasmid

Nrf2 overexpression vector was purchased from Genepharma (Shanghai, China). Overexpression plasmid (pcDNA3.1-Nrf2) was transfected into logarithmically growing LN229 and T98G cells using Lipofectamine 3000 (Thermo Fisher Scientific). After transfection, cells were collected and the increased expression level of Nrf2 was confirmed by western blot assays.

### Transfection of interfering RNA (siRNA)

The si-RNA targeting Nrf2 and its negative control siRNA (si-control) were obtained from GenePharma (Shanghai, China). The sequences of the siRNAs are shown in Additional file 1: Table S2. Transfection was conducted according to Lipofectamine 3000 (Thermo Fisher Scientific) instructions.

### The xenograft tumor model was performed

LN229 cell suspension (4 × 10^6^ cells/100 μL) was subcutaneously injected into the axilla of 6-week-old nude mice (Sibeifu Biotechnology Co., Ltd, Beijing, China). When the tumor volume was visible (7 days after inoculation), all animals were randomly divided into four groups, each with 6 mice (3 females, 3 males), which were the control group (saline), juglone group (200 mg/kg/day), Fer-1 (0.8 mg/kg/day) group, and Fer-1 (0.8 mg/kg/day) + juglone (200 mg/kg/day) group, receiving abdominal injections every other day (3 times a week), with tumor size measured every 3 days, one mouse from each group died early during experimental procedure. 17 days after the treatment, the mice were euthanized and the tumors were removed. Then, subsequent Western blot analysis and immunohistochemical experiments were conducted with tumor tissues obtained from different pharmaceutical groups. This animal experiment was approved by the Ethical Committee of China Medical University (Approval No.: CMUXN2023016; Approval Date: June 28, 2023).

### Data analysis

Statistical analyses were performed using GraphPad Prism9 (GraphPad software). All experiments were performed at least three independent times and results were expressed as the mean ± SD (standard deviation). p < 0.05 was considered statistically significant (*p < 0.05, **p < 0.01, ***p < 0.001).

## Results

### Juglone significantly inhibits the proliferation of GBM cells and induces cell apoptosis

**Fig. 1:**
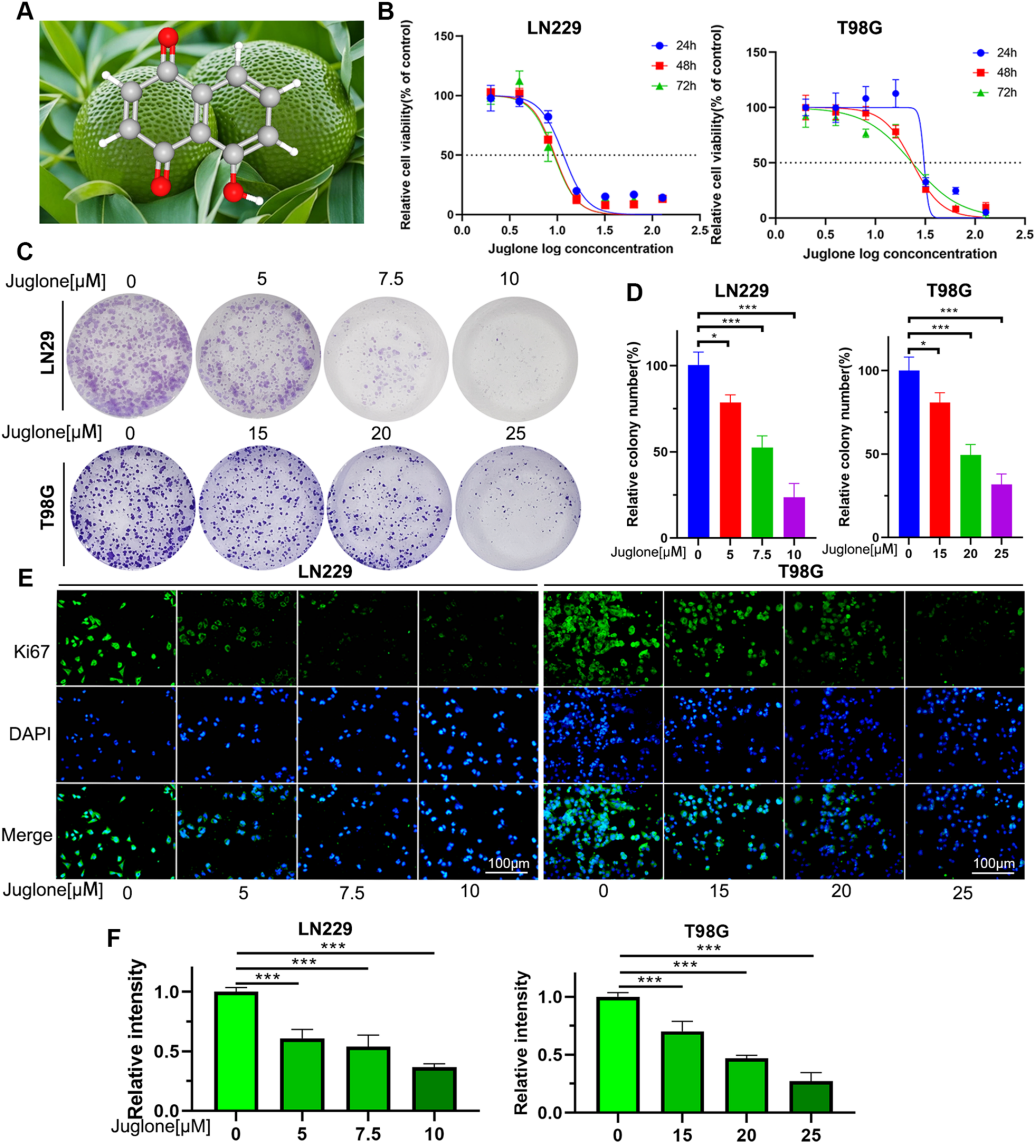
Juglone inhibits cell proliferation and promotes cell apoptosis in GBM cells. **A** The 3D chemical structures of juglone. **B** The CCK-8 assay was used to assess the cell viabilities of LN229 and T98G cells exposed to varying concentrations and durations of juglone exposure. **C**, **D** The LN229 and T98G cells were treated with different concentrations of juglone for 14 days, the clone formation and quantitative analyses were examined. **E**, **F** The LN229 and T98G cells were treated with juglone for 24 h, the Ki67 positivity rates in drug groups at different concentrations by immunofluorescence detection. *p < 0.05;***p < 0.001

To determine the anti-tumor effect of juglone on GBM cells, we treated two types of GBM cells, LN229 and T98G cells, with different concentrations of juglone. Its chemical structure is shown in Fig. 1A. We used the CCK-8 method to determine that juglone significantly inhibited GBM cells proliferation in a time‐dependent manner. The IC50 values of juglone are 11.62 μM (24 h), 9.417 μM (48 h), and 9.214 μM (72 h) in LN229 cells, and 28.80 μM (24 h), 23.52 μM (48 h) and 23.45 μM (72 h) in T98G cells, respectively. Therefore, we chose the IC50 value of juglone at 24 h for subsequent experiments. The drug concentration gradients were set at 5 μM, 7.5 μM, 10 μM (LN229) and 15 μM, 20 μM, 25 μM (T98G), respectively, for subsequent experiments (Fig. 1B). Compared with the control group, after the treatment with juglone, the cell colonies shrank, and the number of cells decreased in a dose-dependent manner (Fig. 1C, D), validating the inhibitory effect of juglone on the proliferation of GBM cells. Further Ki67 immunofluorescence detection results showed that in LN229 and T98G cells, the fluorescence intensity of Ki67 in the two types of glioma cells increased with the increase in drug concentration when induced by juglone (Fig. 1E, F). In summary, the above results indicated that juglone markedly inhibited cell proliferation and enhanced apoptosis in GBM.

### Juglone induces ferroptosis in GBM cells

**Fig. 2:**
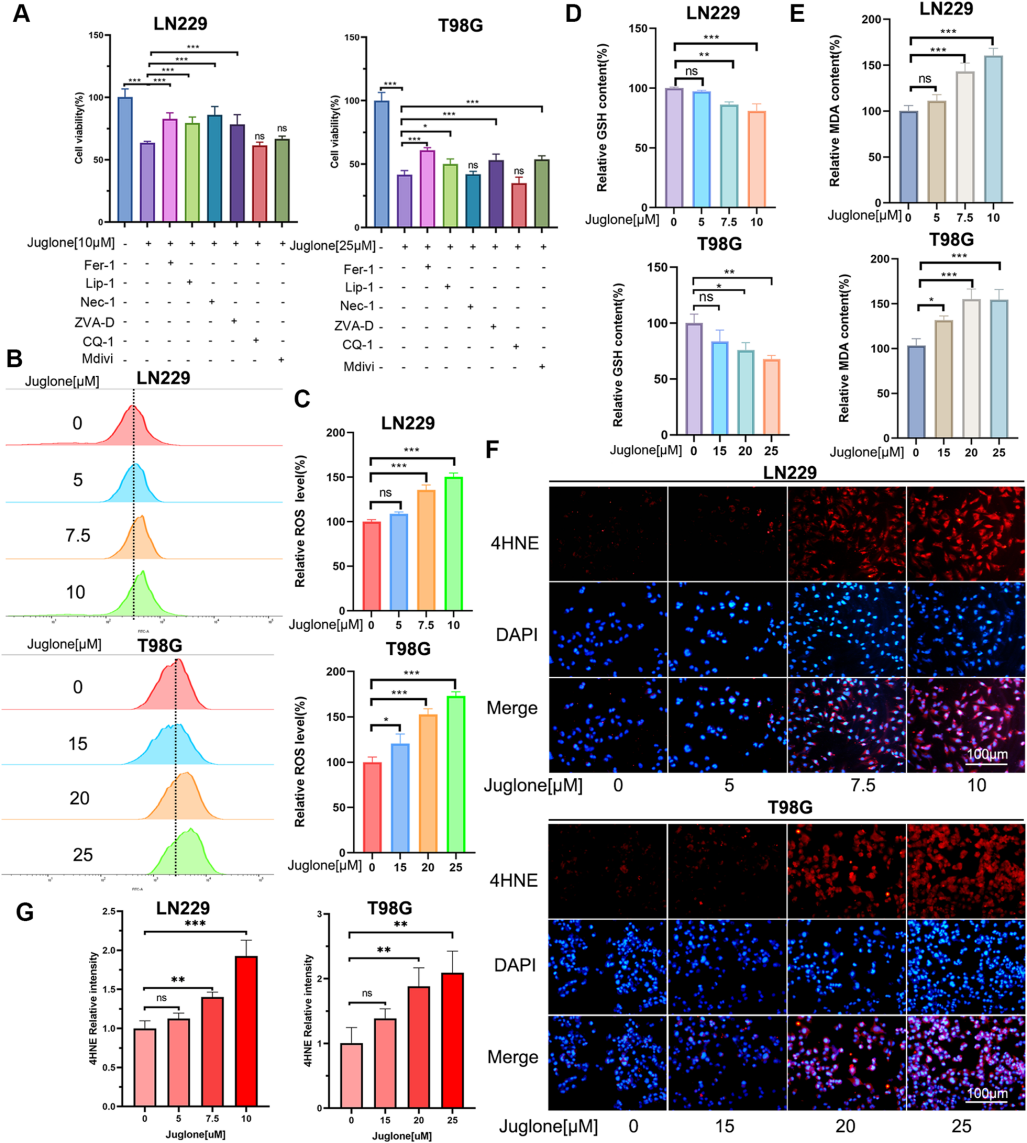
Juglone induces cell death phenotypic changes in GBM cells. **A** LN229 and T98G cells were pretreated with inhibitors such as Fer-1 (4 μM), Lip-1 (0.1 μM), Nec-1 (10 μM), Z-VAD (25 μM), CQ-1(10 μM) or Mdivi(20 μM) for 1 h and then co-treated with juglone for 24 h. The cell viability was determined through a CCK-8 assay. **B**, **C** LN229 and T98G cells were treated with different concentrations of juglone for 24 h. The ROS levels of each group of cells were detected and quantitatively analyzed through flow cytometry. **D**, **E** The levels of GSH and MDA in LN229 and T98G cells were measured under the effect of different concentrations of juglone. **F**, **G** After 24 h of treatment with different concentrations of juglone, the 4HNE level in LN229 and T98G cells was detected and quantitatively analyzed through immunofluorescence. *Fer-1* ferrostatin-1, *Lip-1* liproxstatin-1, *Nec* necrostatin-1, *Z-VAD* Z-VAD-FMK, *CQ-1* chloroquine, *Mdivi* Mdivi-1, *GSH* glutathione, *MDA* malonaldehyde; *p < 0.05; **p < 0.01; ***p < 0.001; ns, no statistical significance

**Fig. 3:**
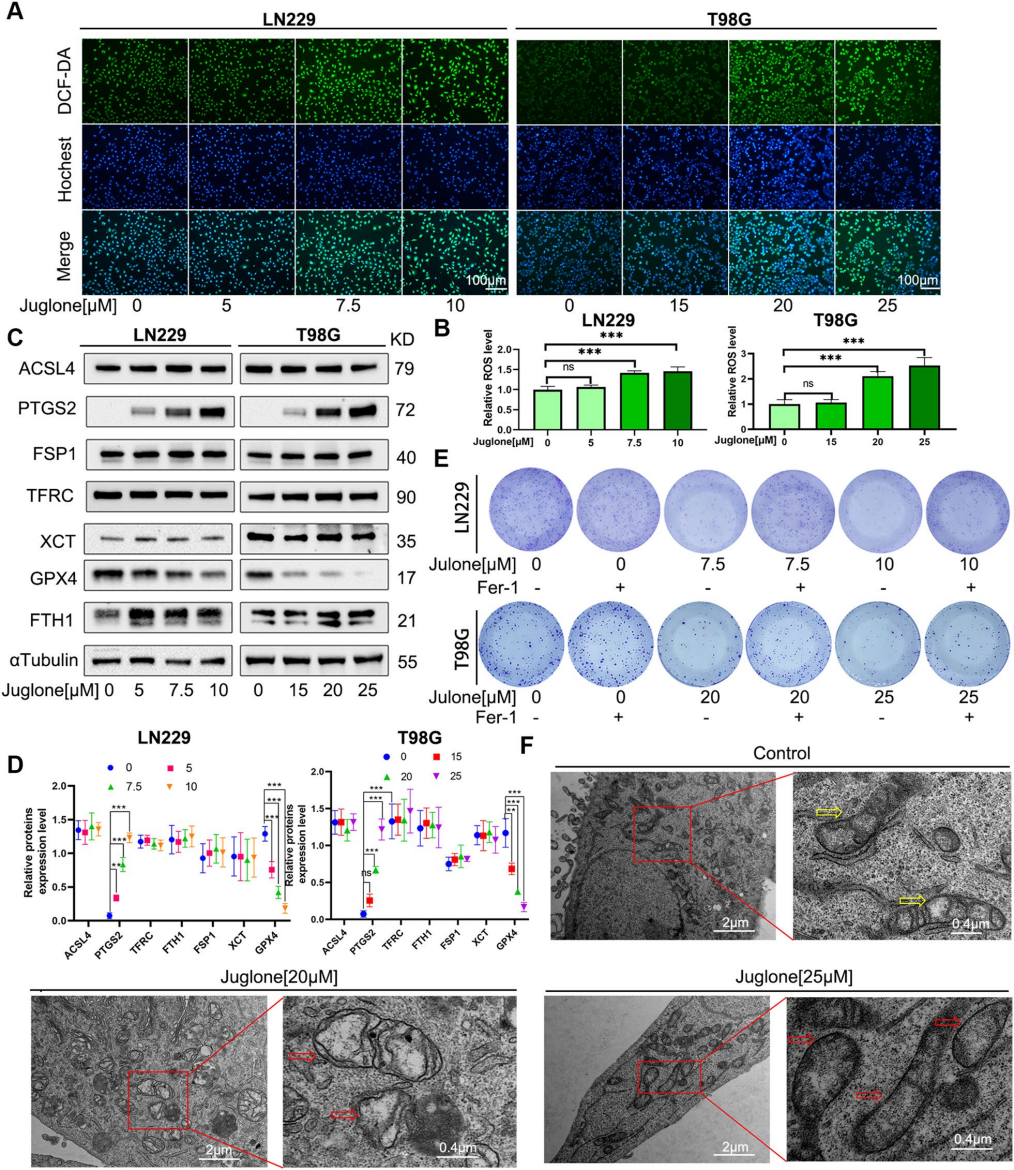
Juglone induced the ferroptosis in GBM cells. **A**, **B** The fluorescence dye DCFDA has been used to measure the ROS level in LN229 and T98G cells and quantitatively analyze the fluorescence intensity. **C**, **D** The expression of key proteins in the ferroptosis pathway in LN229 and T98G cells under the action of juglone through WB experiments. **E** LN229 and T98G cells with or without Fer-1 and observed for about 14 days under different juglone concentrations for cell clone formation. **F** Mitochondrial morphology in different groups was observed using transmission electron microscopy in T98G cells. Yellow arrows indicated normal mitochondrial morphology, and red indicated damaged mitochondrial morphology after juglone induction

We further explore the role of ferroptosis induced by juglone in GBM cells. To examine the effects of different cell death inhibitors, LN229 and T98G cells were cotreated with juglone and various inhibitors of cell death for 24 h.CCK-8 experiments show that Fer-1 and Lip-1, inhibitors of ferroptosis, can significantly reverse the inhibitory effect of juglone on LN229 and T98G cell activity (Fig. 2A), implying that ferroptosis is the major mechanism through which juglone induces death in GBM cells. Characteristics of ferroptosis are accumulation of ROS and lipid peroxides. By using flow cytometry technique to measure ROS in LN229 and T98G cells, we found that Juglone can dose-dependently enhance the accumulation of ROS in GBM cells (Fig. 2B, C). At the same time, immunofluorescence experiments also yielded the same result, namely, juglone significantly enhanced the green fluorescence intensity of GBM cells (Fig. 3A, B). As glutathione (GSH) consumption is a necessary process for ferroptosis, through GSH detection, we verified the predicted result that juglone can reduce the content of GSH (Fig. 2D). MDA and 4HNE are by-products of lipid peroxidation, and our results show that an increase in juglone concentration can trigger an increase in these two peroxides, with a significant statistical difference compared with the control group (Fig. 2E–G). We measured the expression of several key proteins relating to ferroptosis. WB experiments showed that in LN229 and T98 cells treated with juglone, the expression level of PTGS2 increased and the expression level of GPX4 decreased (Fig. 3C, D). Similarly, plate cloning experiments show that Fer-1 can reverse the reduction in the number of T98 and LN229 cell colonies induced by juglone (Fig. 3E, Additional file 1: Fig. S1). To further confirm whether juglone induces ferroptosis in GBM cells, we used transmission electron microscopy to detect morphological changes in mitochondria in T98G cells after 24 h of juglone treatment. The results show that when the juglone concentration is 20 μM, some mitochondrial membranes are significantly thickened, the mitochondria shrunk, there is a decrease or disappearance of mitochondrial cristae, and some mitochondria are swollen. As the drug concentration increased to 25 μM, more mitochondria shrank and the mitochondrial membrane ruptured, which is consistent with the characteristics of mitochondrial damage in ferroptosis (Fig. 3F). Simultaneously, after 24 h of juglone treatment, RNA was extracted from T98G cells and transcriptome sequencing was carried out. The results of the differential gene analysis obtained by comparing with the blank control group and the ferroptosis-related genes dataset from FerrDb were incorporated into the R package “clusterProfiler” for GSEA enrichment analysis. The results show a statistically significant difference (P < 0.05), suggesting a potential association between gene changes under the effect of juglone and the ferroptosis pathway. The overall results show that ferroptosis is the main form of cell death induced by juglone in GBM cells.

### Juglone promotes p38 MAPK phosphorylation to regulate the Nrf2/GPX4 axis

**Fig. 4:**
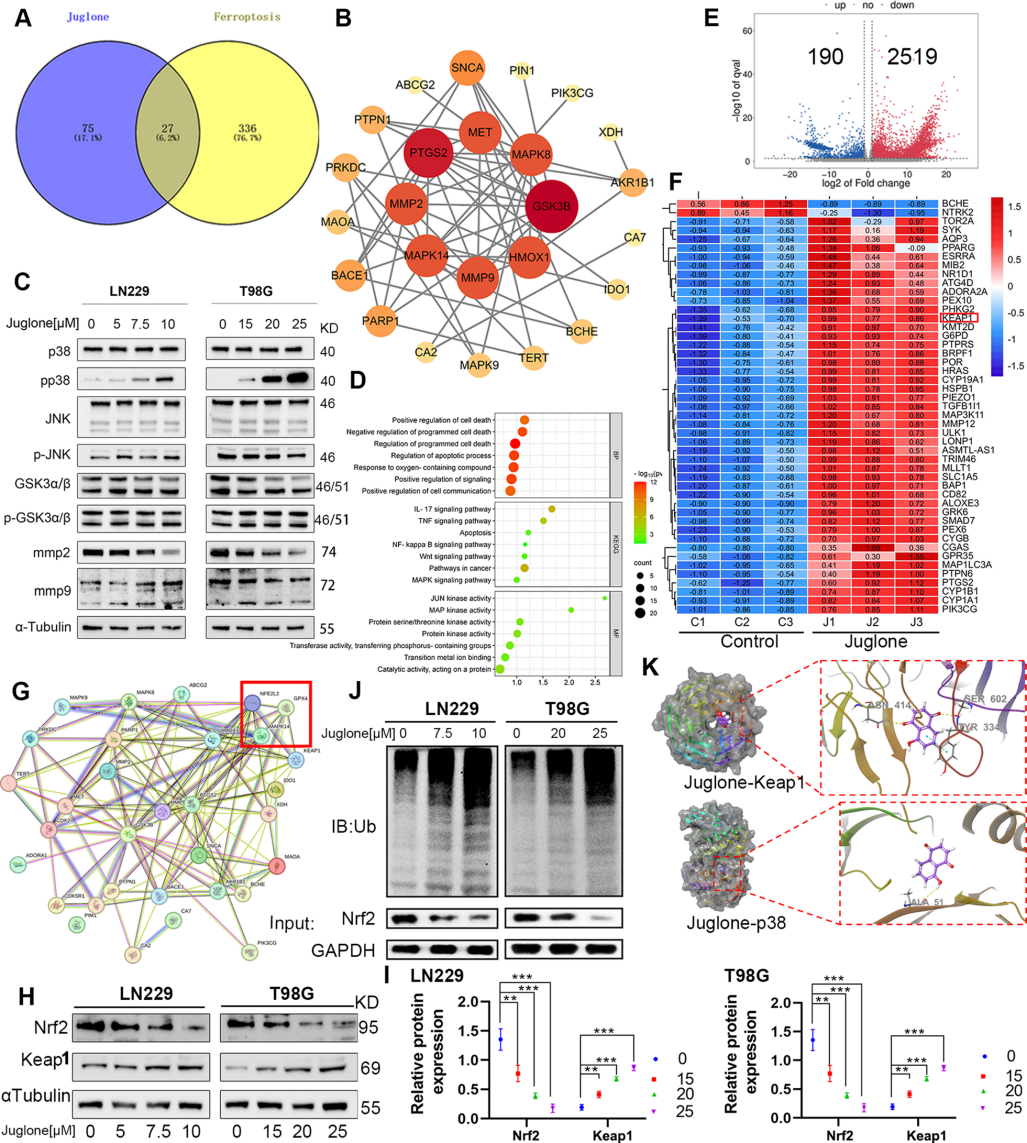
Juglone induced ferroptosis by targeting p38MAPK. **A** Venn diagram shows the intersection of juglone's drug gene targets and ferroptosis related targets. **B** The STRING database was used to analyze the interactions of 27 co-targets in **A**, and the entire network was identified and visualized through Cytosscape. (The color and size of circles represent the significant correlation and the degree of association, respectively. The larger the circle, the more number of enrichment, and the redder the color, the more significant the difference). **C** The key genes were verified using WB experiments. **D** GO and KEGG pathway enrichment analysis were performed on the 27 co-targets. **E** Volcano plot shows the distribution of differential expression genes between the control group and the juglone group through transcriptomics sequencing. **F** Heat map shows the intersection of significantly different genes obtained based on sequencing results and ferroptosis-related genes. **G** Using the STRING database to build a functional protein association network of predicted targets related to juglone, p38 and GPX4. **H, I** WB assay was used to detect Nrf2 and Keap1 protein expression levels with different concentrations of juglone. **J** After immunoprecipitation with anti-Nrf2 antibody, Nrf2 ubiquitylation were assayed by WB analysis. **K** The molecular docking was used to predict the interaction between Juglone and Keap1 and p38MAPK respectively. **p < 0.01; ***p < 0.001

Using the Swiss Target Prediction database, 102 genes for potential drug target of juglone were selected. The intersection of the predicted drug targets and the ferroptosis-related genes from FerrDb database identified 27 key targets (Fig. 4A), which were analyzed using the String database and visualized through the Cytoscape software to identify the entire protein–protein interaction network (Fig. 4B). Through GO annotation, proteins were sorted into two main categories (biological processes and molecular functions). GO and KEGG enrichment analysis suggested that juglone induced ferroptosis is related to the MAPK family activity (Fig. 4D). Meanwhile, we selected the targets with the highest correlation and combined with existing literature, the expression of these key targets was verified through WB experiments. The results revealed that the expression levels of p-p38 and mmp2 significantly increased with the increase of drug concentration (Fig. 4C, Additional file 1: Fig. S3). We introduced GPX4 into the STRING network, as shown in Fig. 4G, and it indicated that p38 might interact with the Nrf2 -GPX4 axis, suggesting that this pathway may play a major role in juglone-induced ferroptosis. Transcriptome sequencing showed that, compared to the blank control group, juglone treatment resulted in significant upregulation of 2519 genes and significant downregulation of 190 genes (Fig. 4E). As the heatmap displayed within the intersection results of the FerrDb database showed, The gene expression levels Keap1 was significantly higher in the juglone treatment group (Fig. 4F). It is known that Keap1 is a negative regulator of Nrf2. WB analysis confirmed that with the increase in drug concentration of juglone, the expression levels of Keap1 protein increased and those of Nrf2 protein decreased significantly (Fig. 4H, I), consistent with this result, Nrf2 in both nucleus and cytoplasm decreased (Additional file 1: Fig. S4). Considering that Nrf2 is mainly degraded in the cell through ubiquitination, the immunoprecipitation experiment was conducted to detect the ubiquitination modification of Nrf2 protein in different juglone concentrations. The results showed that the level of Nrf2 ubiquitination increased with the concentration of juglone (Fig. 4J). We then conducted docking studies to verify the binding ability of juglone with p38 and Keap1, respectively. Juglone penetrates deep into the active pocket of p38 protein, forming a hydrophobic interaction with the residues ALA51, VAL38, PHE169, LEU167, ILE84, etc., of the p38 protein, and forming a hydrogen bond with the residue ALA51. Binding energy calculation showed that the binding energy of juglone-P38 was − 19.77 kcal/mol, indicating that juglone stably binds to p38. Similarly, juglone binds to the surface of the active pocket of Keap1 protein, forming a hydrophobic interaction with the residues LEU139, ALA138, LEU134, LEU110, LEU137, etc. of Keap1 protein, forming a hydrogen bond with the residues ASN414, SER602, and forming a π–π bond with the residue TYR334. The binding energy of Juglone to Keap1 was − 26.49 kcal/mol, indicating that juglone stably binds to p38 (Fig. 4K).

### Inhibition of p38 phosphorylation or activation of Nrf2 reverses ferroptosis induced by juglone

**Fig. 5:**
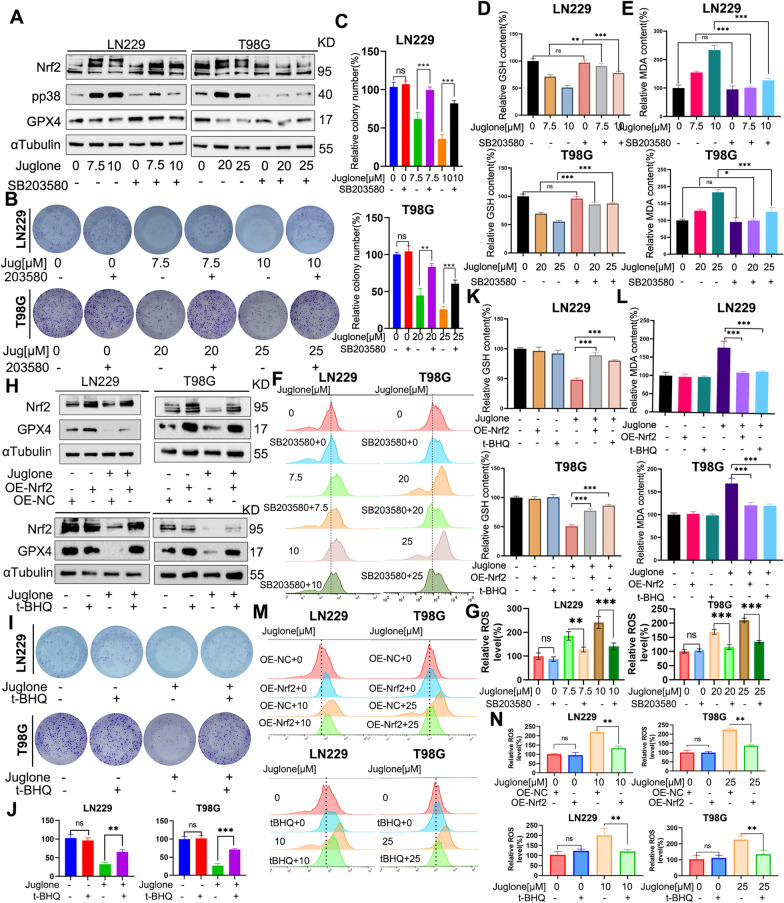
Juglone activates p38MAPK and regulates the Nrf2/GPX4 axis in GBM cells. **A** WB experimental analysis of the expression levels of p-p38, Nrf2, and GPX4 in LN229 and T98G cells incubated for 24 h with different concentrations of juglone combined or not combined with SB203580 (10 µM, preprocessed for 1 h). **B, C** Images and statistical analysis results of plate clone formation of LN229 and T98G cells incubated for about 14 days with different concentrations of juglone combined or not combined with SB203580 (10 µM, preprocessed for 1 h). **D, E** Determination of MDA and GSH content in LN229 and T98G cells after 24 h of action with different concentrations of juglone combined or not combined with SB203580 (10 µM, preprocessed for 1 h). **F, G** Determination of ROS levels in LN229 and T98G cells after 24 h of action with different concentrations of juglone combined or not combined with SB203580 (10 µM, preprocessed for 1 h) based on flow cytometry analysis. **H**. Protein expression levels of GPX4 and Nrf2 in LN229 and T98G cells after 24 h of treatment with juglone in combination with t-BHQ (20 µM, preprocessed for 1 h) or overexpression of Nrf2, based on WB experiment. **I**, **J** Status of cell clone formation and statistical analysis under the action of juglone combined or not combined with t-BHQ (20 µM, preprocessed for 1 h) for about 14 days. **K**, **L** Determination of MDA and GSH content in LN229 and T98G cells after 24 h of treatment with juglone in combination with t-BHQ (20 µM, preprocessed for 1 h) or overexpression of Nrf2. **M**, **N** Detection and statistical analysis of ROS levels in LN229 and T98G cells after 24 h of treatment with juglone in combination with t-BHQ (20 µM, preprocessed for 1 h) or overexpression of Nrf2 by flow cytometry analysis. *p < 0.05; **p < 0.01; ***p < 0.001; ns, no statistical significance

Next, we investigated whether inhibition of p38 kinase phosphorylation would reverse the inhibitory effect on Nrf2 expression and its downstream GPX4 expression induced by juglone. Through WB analysis, we observed that the p38 phosphorylation inhibitor SB203580 inhibited the downregulation of Nrf2 and GPX4 in LN229 and T98 cells induced by juglone (Fig. 5A). Meanwhile, Plate clone formation experiment showed that SB203580 partially rescued the death of LN229 and T98G cells induced by juglone (Fig. 5B, C). In addition, the consumption of GSH and accumulation of MDA were also rescued by SB203580 (Fig. 5D, E), and SB203580 also rescued the ROS accumulation induced by juglone (Fig. 5F, G).

To further verify whether the Nrf2/GPX4 pathway is involved in the ferroptosis of GBM induced by juglone, we added the Nrf2 protein agonist t-BHQ or used the Nrf2 overexpression vector to transiently transfect LN229 and T98G cells. As shown in Fig. 5H and Additional file 1: Fig. S3, the above treatments confirmed that the increased expression level of Nrf2 protein rescued the expression of GPX4 from the juglone-induced reduction. In addition, treatment with t-BHQ activated Nrf2 expression, partially rescued the growth inhibition of LN229 and T98G cells induced by juglone in the plate clone experiment (Fig. 5I, J). Consistent with this, the accumulation of ROS was successfully eliminated by overexpression of Nrf2 and activation of t-BHQ (Fig. 5M, N). Similar results were also observed for the levels of GSH and MDA (Fig. 5K, L). In order to further explore the effect of juglone on Nrf2 in GBM cells, we designed two si-RNAs targeting Nrf2. The experimental results showed that knocking down Nrf2 enhanced the cytotoxic effect of juglone on T98G cells, and at the same time, knocking down Nrf2 further increased the accumulation of MDA and the consumption of GSH induced by juglone in T98G cells. These results indicate that the Nrf2/GX4 axis is involved in the ferroptosis process induced by juglone, and juglone can induce GBM cells ferroptosis by inhibiting the Nrf2/GPX4 pathway through activating p38 phosphorylation.

### Juglone inhibits ferroptosis induced by the Nrf2/GPX4 axis in a GBM xenograft nude mice model

**Fig. 6:**
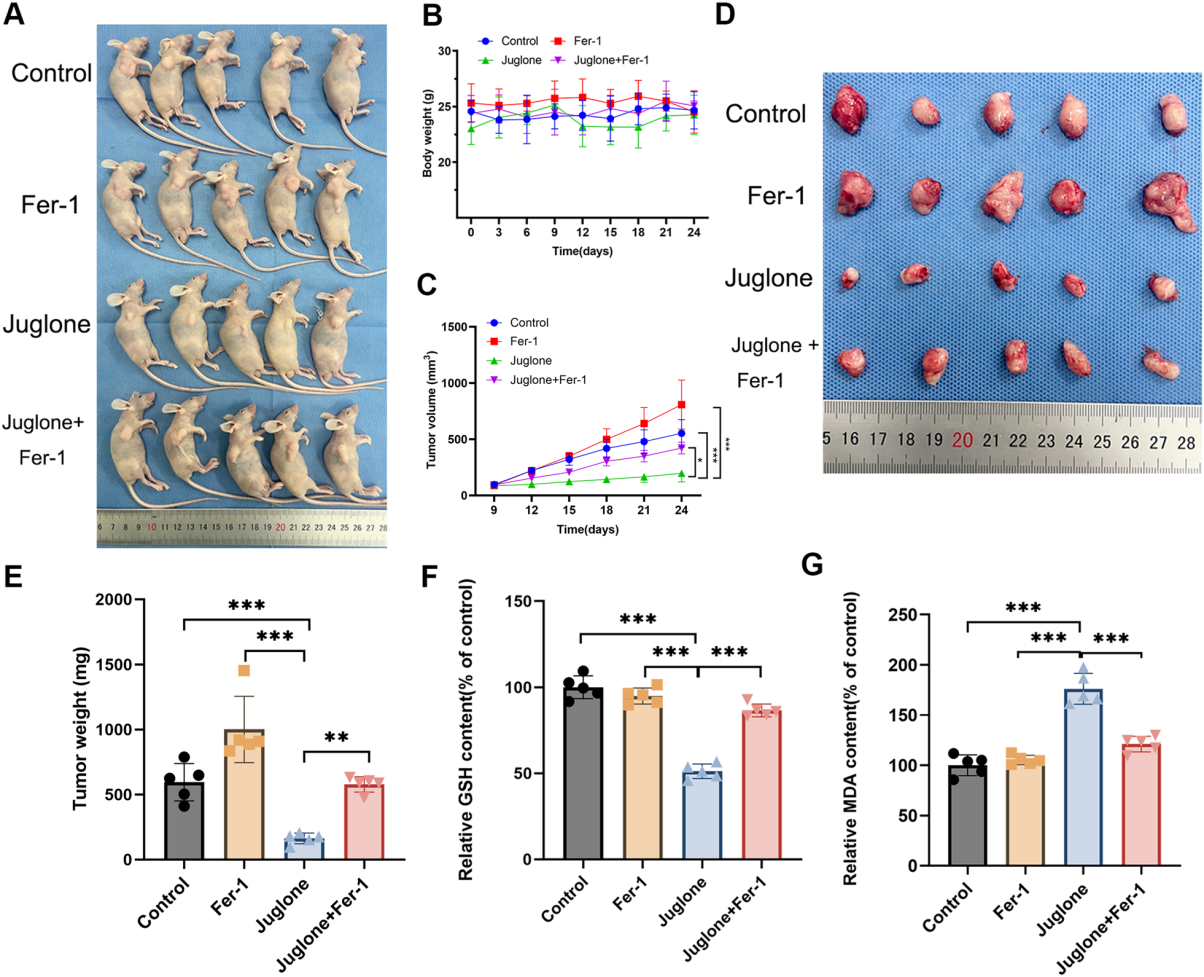
Fer-1 successfully reversed the tumor suppression of juglone in vivo. The condition of Xenograft model in nude mice treated with intraperitoneal injection of either physiological saline, juglone (1 mg/kg), Fer-1 (10-mg/kg/d), or Fer-1 (10-mg/kg/d) + juglone (1 mg/kg) respectively. **A** Tumor-bearing mice in each group at 24th days. **B**, **C** The changes in body weight and tumor volume of nude mice are shown in each group. **D** The representative images of burdened tumors in each group. **E**–**G** Measure the weight of the subcutaneous tumor removed on the 24th day and the levels of MDA and GSH in the tissue. ***p < 0.001

**Fig. 7:**
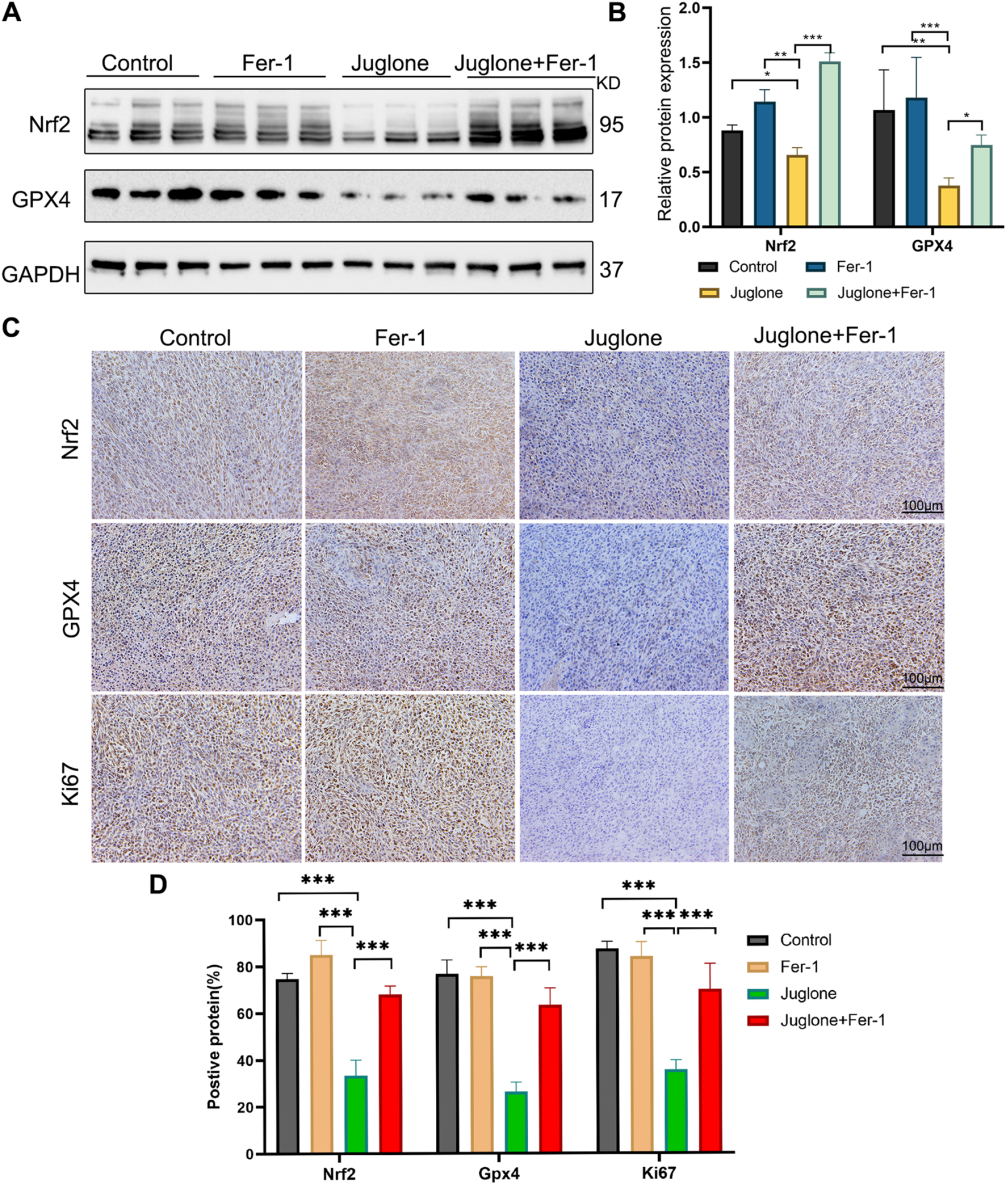
Juglone enhances ferroptosis by inhibiting the Nrf2/GPX4 axis in vivo. **A, B** The protein expression levels of Nrf2 and GPX4 were quantified by western blot analysis. **C, D** The expression level of Nrf2, GPX4 and Ki67 in the tissues was detected by immunohistochemical staining. *p < 0.05; **p < 0.01; ***p < 0.001

**Fig. 8:**
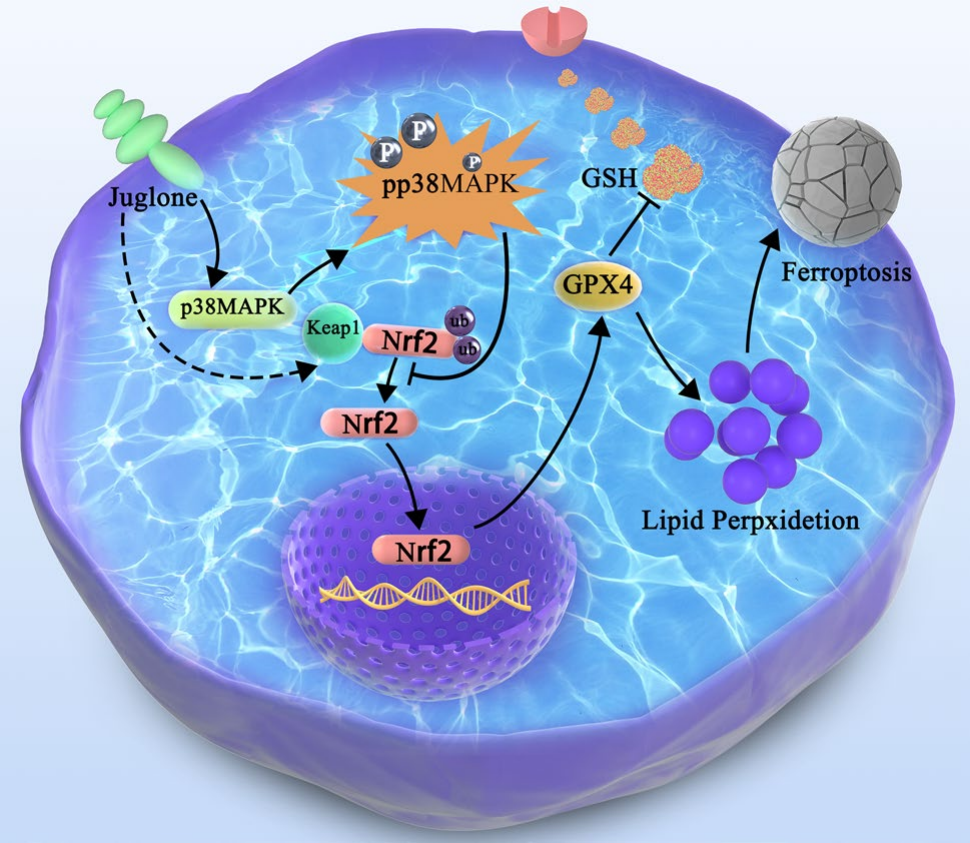
Graphical summary for the underlying mechanism of juglone induces ferroptosis in GBM cells by activating p38 MAPK phosphorylation and inhibiting the Nrf2/GPX4 axis

For an in-depth understanding of juglone’s anti-tumor in vivo, we chose to establish xenograft tumor model of nude mice and administer the drug every other day by intraperitoneal injection according to the experimental scheme. Although we observed that juglone treatment did not significantly affect the weight of the mice (Fig. 6B), we still observed a significant reduction in tumor volume and weight in the treatment group. Notably, the reduction in tumor volume and weight were reversed by the ferroptosis inhibitor Fer-1 (Fig. 6C–E). In our study, we also observed a similar reversal of the MDA and GSH levels in subcutaneous tumors in mice treated with juglone (Fig. 6G). To assess the effects of juglone more comprehensively, we performed further WB and immunohistochemical experiments. The results show that juglone significantly inhibits the expression of Ki67, GPX4, and Nrf2, and this inhibition is significantly reversed by the injection of Fer-1 (Fig. 7A–D). Thus, it can be seen that juglone inhibits tumor growth in vivo by inducing ferroptosis. Based on all these results, we have proposed a mechanistic model to explain the potential role of juglone in GBM cells (Fig. 8).

## Discussion

Glioblastoma (GBM), known to be highly heterogeneous, is a prevalent malignant tumor in the central nervous system [40]. It is characterized by invasive nature, short overall survival, and poor prognosis. Traditional therapies, encompassing radiation and chemotherapy, render limited effects on GBM, making its treatment a considerable challenge [41–43]. Thus, the need for new treatment strategies is imperative. Iron metabolism is pivotal in carcinogenesis cellular metabolism and growth of tumor cells. Consequently, ferroptosis, a unique form of cell death, could offer a potential anti-tumorigenic strategy in GBM [44, 45].

Distinct from apoptosis, necrosis, or autophagy, ferroptosis involves oxidative stress-related cell death and mediated by iron-dependent lipid peroxidation accumulation [46]. Given that the central nervous system which is rich in polyunsaturated fatty acids (PUFA), the peroxidation of PUFA could induce ferroptosis [47]. Current research proposes that ferroptosis inducers could boost the chemotherapeutic effects on GBM, hinder tumor resistance to chemotherapy, and impede intracranial metastasis [48, 49]. Thus, developing ferroptosis inducers is a promising strategy to combat GBM.

Naphthoquinone compounds represent a class of natural substances with a variety of pharmacological effects [50]. They are widely used in various clinical anticancer drugs, such as doxorubicin, daunorubicin, mitomycin, and mitoxantrone [51, 52]. At the same time, many naphthoquinone natural derivatives such as shikonin, resveratrol, plumbagin have shown potential therapeutic value as anticancer drugs [53–55], which is worth further exploration. Juglone, as a natural naphthoquinone compound, its potential against tumors is increasingly being noticed. Juglone has been proven to inhibit the proliferation and migration of tumor cells, cause cell cycle stagnation, and induce cell death. It is worth mentioning that juglone can penetrate the blood–brain barrier and has clear advantages in the treatment of intracranial malignant tumors [56, 57].

Acting as a transcription factor, Nrf2 can regulate a range of antioxidative genes and enzymes to maintain redox homeostasis to avoid cell damage. It directly affects several genes and enzymes implicated in ferroptosis and plays a vital role. Studies have shown that p38 MAPK serves as an upstream molecule of Nrf2 [58]. However, different cells demonstrate varied, sometimes contrary, regulation mechanisms in the activation of p38mapk on Nrf2. Such as Andrographolide induces Nrf2 and HO-1 expression in astrocytes through p38 MAPK [59]. The combination of Cetuximab and RSL activates P38 phosphorylation to inhibit the Nrf2-HO-1 axis-induced ferroptosis, thereby enhancing resistance [60]. Silica nanoparticles (SiNPs) suppress Nrf2 transcription and expression in human umbilical vein endothelial cells (HUVECs) through P38 phosphorylation activation [61].

We reported for the first time in this study that juglone triggers ferroptosis in GBM. We observed that the Fer-1 (ferroptosis inhibitor) effectively rescue the effect of juglone inhibiting clone formation. Consequently, we studied whether juglone could induce ferroptosis in GBM cells specifically. As anticipated, upon application of juglone, ferroptosis-related event phenotypes such as ROS accumulation, GSH exhaustion, MDA overproduction, and significant mitochondrial damage occurred. To elucidate the mechanism of juglone-induced ferroptosis, we employed SwissTargetPrediction for target prediction and confirmed key targets through WB experiments. We discovered increased phosphorylation levels of P38 in the MAPK family. GO enrichment analysis demonstrated that juglone-induced GBM ferroptosis relates to MAPK activity, after which we detected the P38-NRF2-GPX4 signaling pathway. Furthermore, transcriptome analysis suggested that juglone might elevate the NRF2’s negative regulatory protein Keap1 expression. We confirmed, through a series of in vitro and in vivo functional experiments, that juglone induces ferroptosis in GBM cells by activating P38 phosphorylation and downregulating the Nrf2-GPX4 pathway, thereby inhibiting oxidative stress. But we still need to study further the extent to which Keap1 is involved in regulating the down-regulation of Nrf2 induced by juglone. After the application of juglone, we observed a decrease in mmp2 expression. Given that mmp2 is necessary for promoting tumor invasion, it potentially accounts for the reduced invasive potential of GBM.

However, implementing juglone-induced ferroptosis in the treatment of glioblastoma presents significant challenges. For instance, the regulation of balance between intracellular iron ions is extremely complex. Interfering with iron metabolism could result in other adverse reactions. Additionally, the body's distribution and metabolism of juglone require further investigation. Overall, juglone shows potential as a GBM inhibitor through inducing ferroptosis. Subsequent research should focus on mechanisms concerning intracellular iron metabolism and drug tolerance for the continued development and optimization of juglone-based GBM treatment.

## Conclusions

In conclusion, juglone could potentially serve as a new ferroptosis inducer and might induce GBM cell death. Our data advocate consideration of juglone as a potential therapeutic compound for GBM. To fully harness the potential of juglone in clinical applications, large-scale animal experiments and prospective multicenter studies are needed.

### Supplementary Information

**Additional file 1.** The original data of this experiment

## Data Availability

All data generated or analyzed during this study are included in this published article.

## Abbreviations

ATCC: American type culture collection
CQ-1: Chloroquine
Fer-1: Ferrostatain-1
Lip-1: Liproxstatin-1
Mdivi: Mdivi-1
Nec-1: Necrostatin-1
Z-VAD: Z-VAD-FMK
DCFDA: 2ʹ,7ʹ-Dichlorodihydrofluorescein diacetate
GSH: Glutathione
MDA: Malonaldehyde
RCSB PDB database: Structural bioinformatics protein data bank
GBM: Glioblastoma
ROS: Reactive oxygen species
