# Supplementary material for: Juglone induces ferroptosis in glioblastoma cells by inhibiting the Nrf2-GPX4 axis through the phosphorylation of p38MAPK
Source: Chin Med. 2024 Mar 22;19:52. doi: 10.1186/s13020-024-00920-2 (PMC10958923; doi:10.1186/s13020-024-00920-2)
Supplement: Supplementary file 1 — Additional file 1. The original data of this experiment [file 13020_2024_920_MOESM1_ESM.pdf]

**Supplementary file**

**Table S1**

| Antibodies | Source | Identifier |
|---|---|---|
| ACSL4 | Abcam | ab155282 |
| PTGS2 | Abcam | ab15191 |
| GPX4 | Abcam | ab125066 |
| Ki67 | Abcam | ab16667 |
| 4HNE | Abcam | ab46545 |
| XCT | Cell Signaling Technology | 12691S |
| FTH1 | Cell Signaling Technology | 4393S |
| p38 | Cell Signaling Technology | 9212S |
| pp38 | Cell Signaling Technology | 9215S |
| JNK | Cell Signaling Technology | 9251S |
| p-JNK | Cell Signaling Technology | 9252S |
| GSK3α/β | Cell Signaling Technology | 5676S |
| p-GSK3α/β | Cell Signaling Technology | 8566S |
| MMP2 | Cell Signaling Technology | 40994S |
| MMP9 | Cell Signaling Technology | 13667S |
| TFRC | Invitrogen | 13-6800 |
| α-Tubulin | HuaBio | ER130905 |
| GAPDH | HuaBio | ET1601-4 |

**Table S2**

| |
|---|
| si-Nrf2-1 sense: 5′-GCAUGCUACGUGAUG AAGATT-3′; |
| antisense: 5′-UCUUCAUCACGUAGCAUG CTT-3′ |
| si-Nrf2-2 sense:5′-UCUUCAUCACGUAGCAUGCTT-3′; |
| antisense: 5′-GCAUGCUAC GUGAUGAAGATT-3′) |

**Fig.S1**

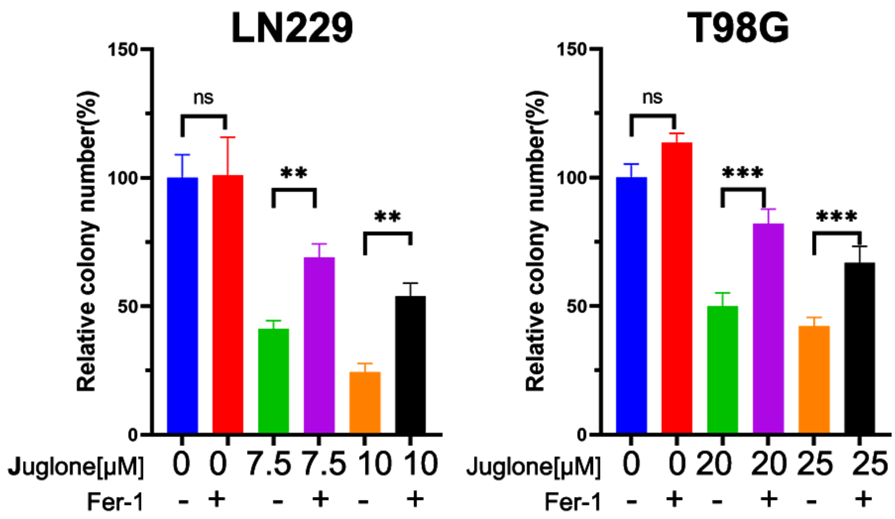


LN229 and T98G cells were treated with Juglone combined or not with Fer-1,the number of cell clones was counted and analyzed. **,$p < 0.01$; ***,$p < 0.001$;ns, no significant difference

**Fig. S2**

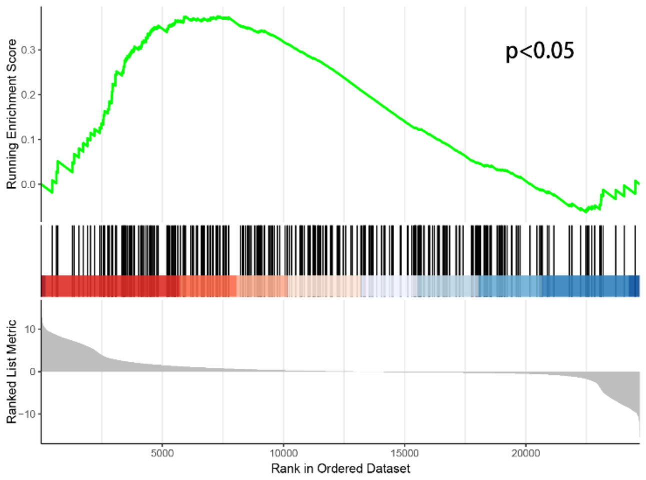


The association between the significantly different genes in the transcriptome and the ferroptosis gene dataset has been identified through the GSEA analysis using the R package“clusterProfiler”.

**Fig. S3**

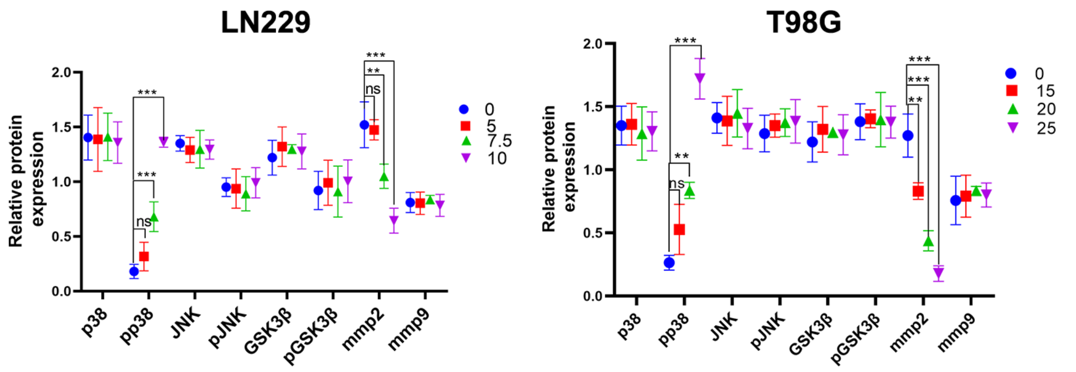


The expression of key proteins selected in LN229 and T98G cells under the action of juglone through WB experiments. **,p < 0.01; ***,p < 0.001;ns, no significant difference.

**Fig. S4**

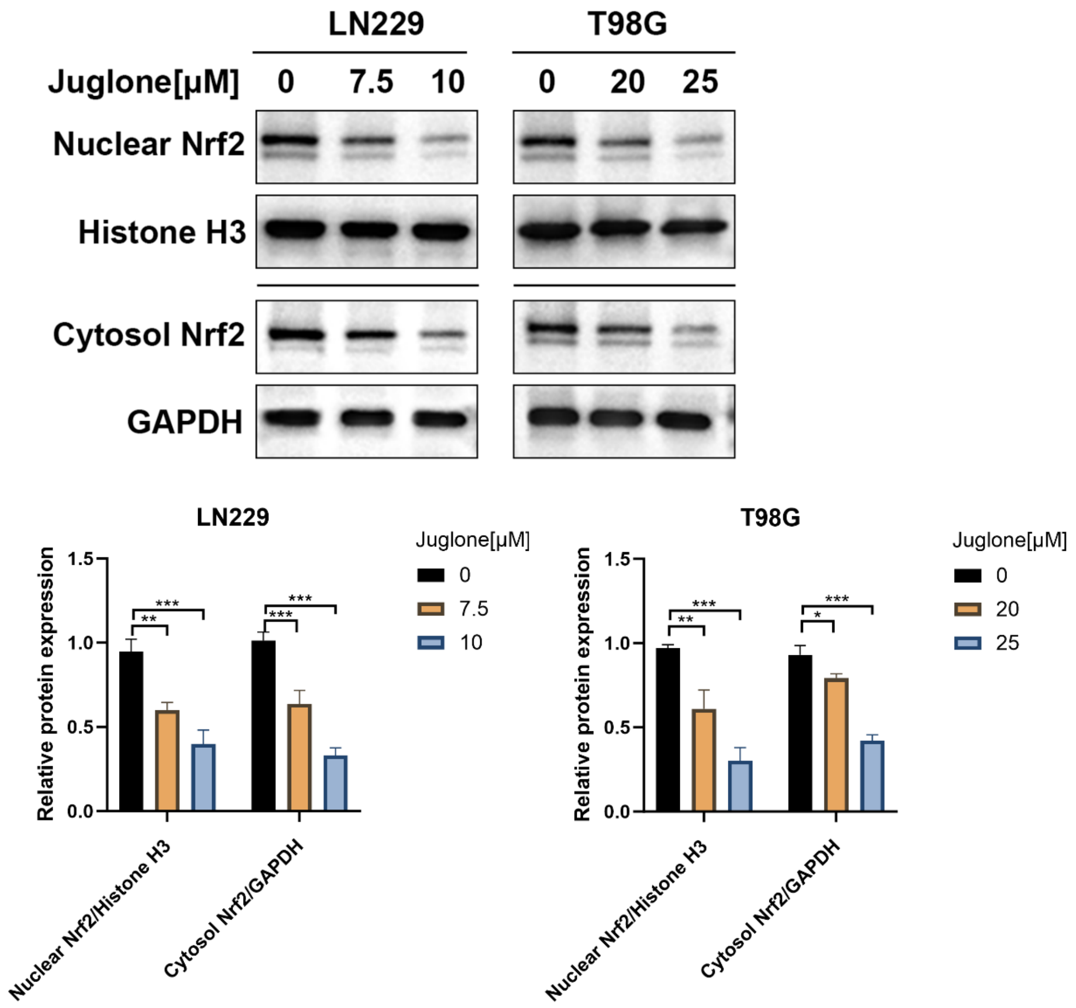


The WB experiment showed the protein expression level of Nrf2 in the cytoplasm and nucleus under the influence of juglone. *,p<0.05; **,p < 0.01; ***,p < 0.001.

**Fig. S5**

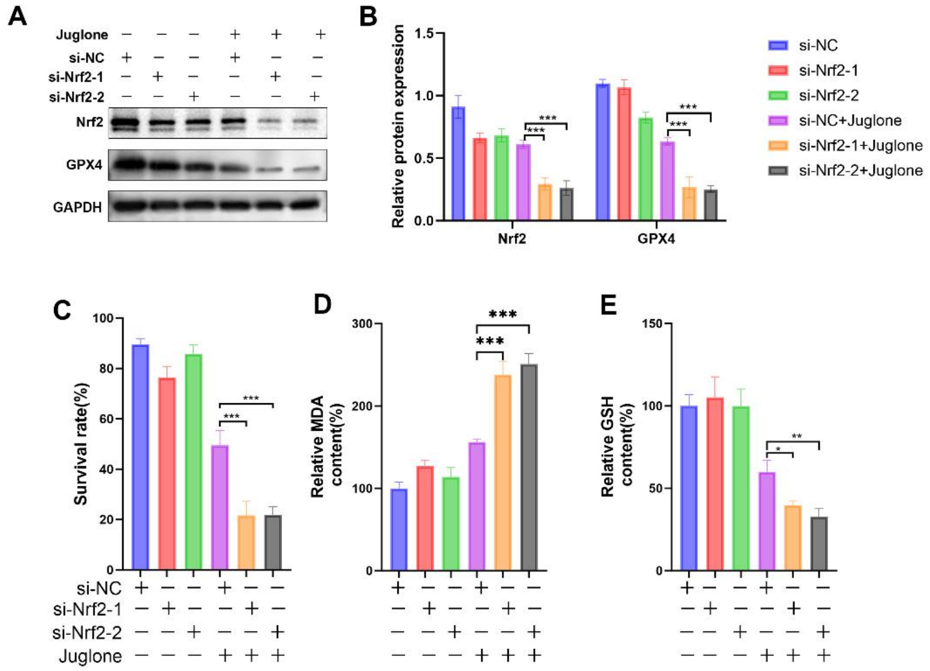


**A.B.** After Nrf2 was knocked down by siRNA, the protein expression levels of Nrf2 and GPX4 in T98G cells were further reduced 24 hours after the treatment with juglone (25μM). **C.** The cell survival viability was assessed by CCK8 assay. **D.E.** The levels of MDA and GSH were measured in Nrf2-knockdown T98G cells pretreated with or without juglone (25μM). *,p<0.05; **,p < 0.01; ***,p < 0.001.

**Fig. S6**

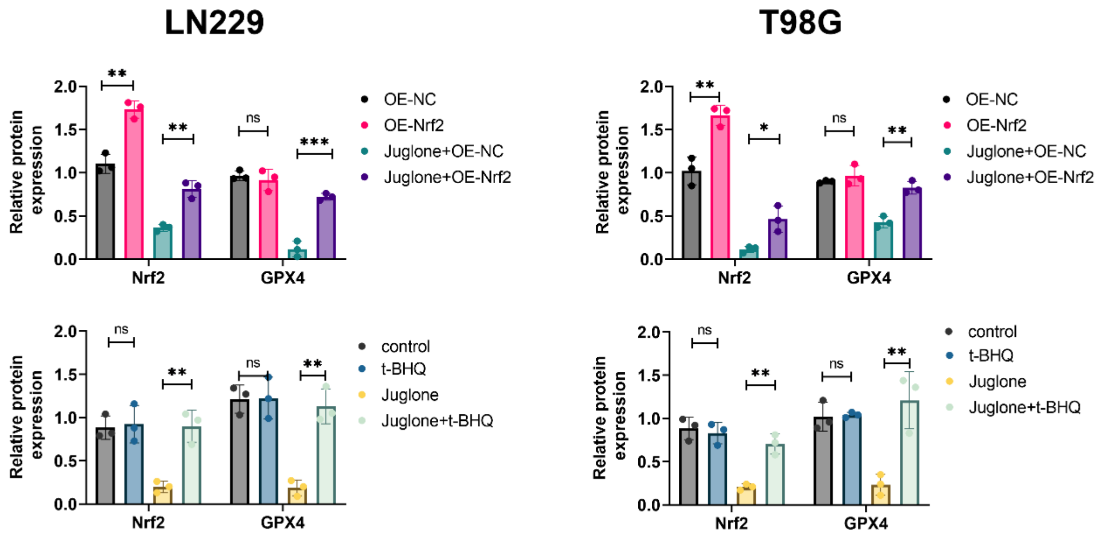


Protein expression levels of GPX4 and Nrf2 in LN229 and T98G cells after 24 hours of treatment with juglone in combination with t-BHQ (20μM, preprocessed for 1h) or overexpression of Nrf2,

based on WB experiment. *,$p<0.05$;**;$p < 0.01$; ***,$p < 0.001$;ns, no significant difference
